# Supplementary material for: Multi‐dataset identification of innovative feature genes and molecular mechanisms in keratoconus
Source: J Cell Mol Med. 2024 Sep 19;28(18):e70079. doi: 10.1111/jcmm.70079 (PMC11412914; doi:10.1111/jcmm.70079)
Supplement: Supplementary file 2 — Material S2. [file JCMM-28-e70079-s002.zip › 7.GSEA analysis/6.SNORA74B/KEGG_TERPENOID_BACKBONE_BIOSYNTHESIS.html]

Details for gene set KEGG\_TERPENOID\_BACKBONE\_BIOSYNTHESIS[GSEA]

|  || Dataset | SNORA74B.group.cls#h\_versus\_l |
| Phenotype | group.cls#h\_versus\_l |
| Upregulated in class | h |
| GeneSet | KEGG\_TERPENOID\_BACKBONE\_BIOSYNTHESIS |
| Enrichment Score (ES) | 0.32473022 |
| Normalized Enrichment Score (NES) | 0.8657454 |
| Nominal p-value | 0.6297872 |
| FDR q-value | 1.0 |
| FWER p-Value | 1.0 |
Table: GSEA Results Summary

  

Fig 1: Enrichment plot: KEGG\_TERPENOID\_BACKBONE\_BIOSYNTHESIS      
 Profile of the Running ES Score & Positions of GeneSet Members on the Rank Ordered List

  

| SYMBOL | TITLE | RANK IN GENE LIST | RANK METRIC SCORE | RUNNING ES | CORE ENRICHMENT || 1 | MVD | na | 7260 | 0.155 | -0.0700 | Yes |
| 2 | IDI2 | na | 7420 | 0.150 | 0.0605 | Yes |
| 3 | MVK | na | 8473 | 0.125 | 0.1427 | Yes |
| 4 | HMGCS2 | na | 10618 | 0.081 | 0.1539 | Yes |
| 5 | PDSS1 | na | 11255 | 0.071 | 0.1991 | Yes |
| 6 | FDPS | na | 11376 | 0.069 | 0.2575 | Yes |
| 7 | IDI1 | na | 12282 | 0.056 | 0.2820 | Yes |
| 8 | PMVK | na | 13166 | 0.045 | 0.2970 | Yes |
| 9 | HMGCR | na | 13502 | 0.042 | 0.3247 | Yes |
| 10 | ACAT2 | na | 15497 | 0.020 | 0.2848 | No |
| 11 | HMGCS1 | na | 16538 | 0.009 | 0.2630 | No |
| 12 | ACAT1 | na | 16541 | 0.009 | 0.2710 | No |
| 13 | PDSS2 | na | 22890 | -0.064 | 0.1452 | No |
| 14 | GGPS1 | na | 23981 | -0.081 | 0.1862 | No |
| 15 | DHDDS | na | 26891 | -0.137 | 0.2258 | No |
Table: GSEA details [plain text format]

  

Fig 2: KEGG\_TERPENOID\_BACKBONE\_BIOSYNTHESIS      
 Blue-Pink O' Gram in the Space of the Analyzed GeneSet

  

Fig 3: KEGG\_TERPENOID\_BACKBONE\_BIOSYNTHESIS: Random ES distribution      
 Gene set null distribution of ES for **KEGG\_TERPENOID\_BACKBONE\_BIOSYNTHESIS**
